# Human adenoviral (HAdV) chronic arthritis expands the infectious spectrum of primary agammaglobulinemia

**DOI:** 10.1186/s12985-022-01905-z

**Published:** 2022-10-31

**Authors:** Grégoire Martin de Frémont, Maud Salmona, François Maillet, Margaux Garzaro, Rémi Bertinchamp, Arthur Simonnet, Linda Feghoul, Guitta Maki, Marie Roelens, Emilie Chotard, Capucine Picard, Eric Oksenhendler, Jérôme LeGoff, David Boutboul

**Affiliations:** 1 Clinical Immunology Department, Hôpital Saint Louis, Assistance Publique-Hôpitaux de Paris (AP-HP), Université Paris Cité, Paris, France; 2 Virology Department, Hôpital Saint Louis, Assistance Publique-Hôpitaux de Paris (AP-HP), Université Paris Cité, Paris, France; 3 Immunology Laboratory, Hôpital Saint Louis, Assistance Publique-Hôpitaux de Paris (AP-HP), Université Paris Cité, Paris, France; 4 Centre d’étude des déficits immunitaires (CEDI), Hôpital Necker, Assistance Publique-Hôpitaux de Paris (AP-HP), Université Paris Cité, Paris, France; 5 Rheumatology Department, Hôpital Lariboisière, Assistance Publique-Hôpitaux de Paris (AP- HP), Université Paris Cité, Paris, France; 6 INSERM U1163, Université Paris Cité, Imagine Institute, Paris, France; 7 Inserm U976, Université Paris Cité, Paris, France

**Keywords:** Adenoviral arthritis, Agammaglobulinemia, Inborn errors of immunity, Metagenomic next generation sequencing

## Abstract

Inborn errors of immunity (IEI) are a heterogeneous entity with an increasing number of late diagnoses. Besides infections, inflammatory manifestations are a growing part of the clinical landscape of IEI. These complications are of unknown causes and often lead to the prescription of immunosuppressive agents that worsen the underlying immune defect. We here report the case of an adult patient diagnosed with chronic Human Adenovirus C-1 arthritis in the setting of primary agammaglobulinemia. Metagenomic next-generation sequencing led to the correct diagnosis and high-dose intravenous immunoglobulins resulted in complete recovery. This observation gives new insights into adenoviral immunity and underlines the importance of metagenomics in the diagnosis of inflammatory manifestations in immunocompromised patients.

**Supplementary Information:**

The online version contains supplementary material available at 10.1186/s12985-022-01905-z.

## Introduction

 Inborn errors of immunity (IEI) are a heterogeneous entity with an increasing number of late diagnoses. B-cell deficiencies are the most common type of IEI, accounting for nearly half of all IEI. This group appears to be highly heterogeneous ranging from agammaglobulinemia with complete absence of serum immunoglobulins (Ig) to selective IgA deficiency or abnormal polysaccharide responses [1, 2]. Agammaglobulinemia is frequently associated with a complete absence of B cells, among which X-linked agammaglobulinemia (XLA or Bruton’s disease), related to deleterious loss-of-function mutations in the *Bruton Tyrosine Kinase (BTK*) gene, is the leading cause. In patients with agammaglobulinemia, bacterial upper and lower respiratory tract infections (RTIs) and bacterial and parasitic digestive infections are frequently observed and can be life-threatening, especially before initiation of immunoglobulin replacement [3]. Acute infectious joint disease is less frequent and usually involved *Streptococcus pneumoniae, Haemophilus influenzae* and *Staphylococcus*. Other bacteria such as *Mycoplasma sp.* and *Ureaplasma sp.* have been described in chronic inflammatory joint disorders [4]. Patients with agammaglobulinemia also display viral susceptibility mainly consisting in chronic enteroviral meningo-encephalitis [3]. We here report the case of an adult male patient diagnosed with chronic Human Adenoviral C-1 (HAdV C-1) inflammatory joint disorder in the setting of agammaglobulinemia. Metagenomic next generation sequencing (MNGS) led to the correct diagnosis and high-dose intravenous Ig therapy was started with rapid and complete recovery.

## Clinical narrative

**Fig. 1:**
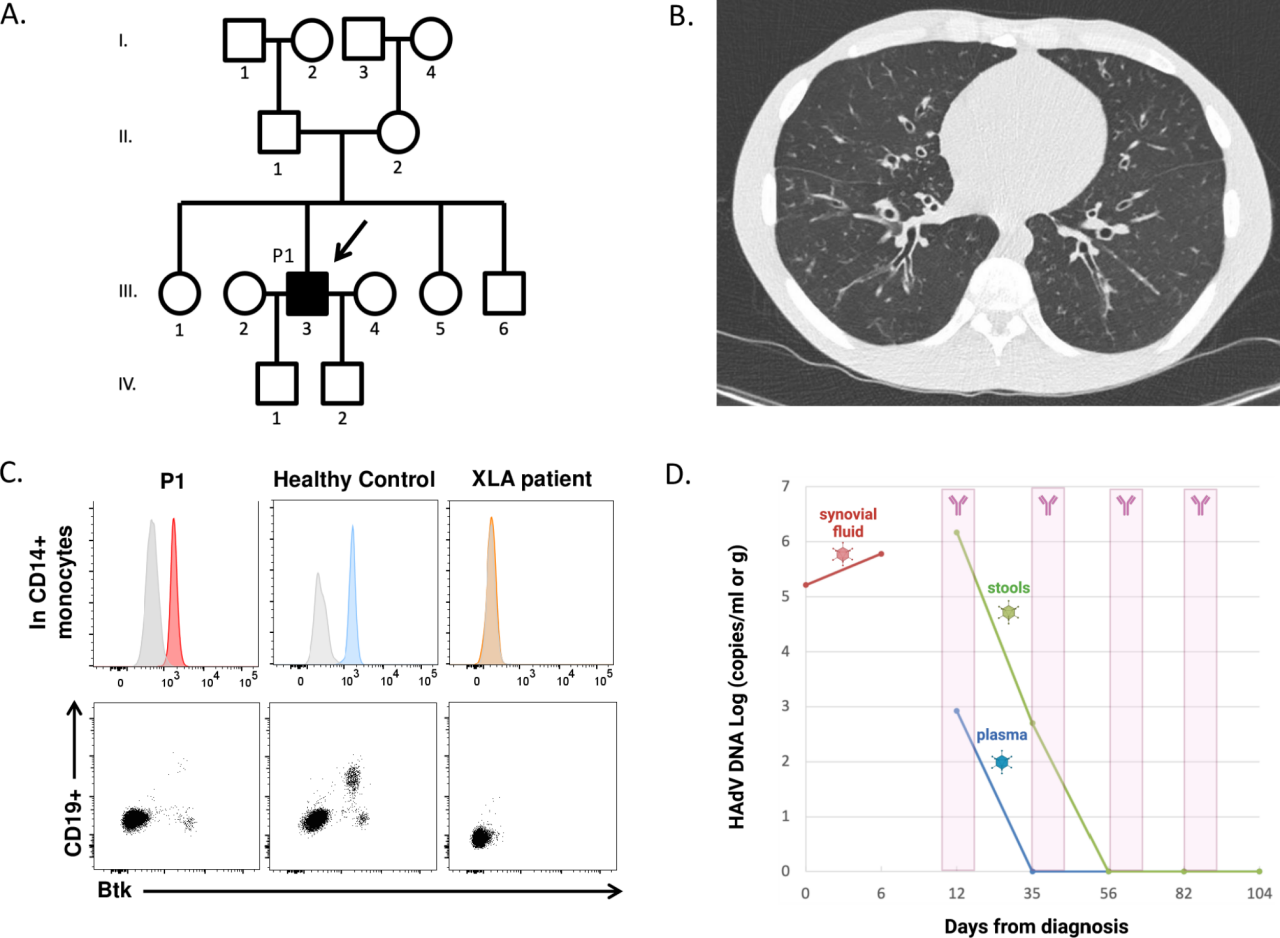
A. Pedigree of the patient (depicted in black, III.3 or P1) with Human Adenoviral polyarthritis B. Chest Computed Tomography of P1 showing diffuse bronchiectasis. C. Bruton’s Tyrosine Kinase (Btk) expression in CD14 + monocytes and in rare CD19 + B-cells of P1 compared to a healthy control and to a patient with X-linked agammaglobulinemia (XLA or Bruton’s disease). D. Kinetics of HAdV DNA in synovial fluid (red), stools (green) and plasma (blue). Pink area correspond to timepoints of IVIg infusion

A 33-year-old North African male born from healthy and non-consanguineous parents was admitted to the Rheumatology department for the exploration of a chronic and non-erosive polyarthritis, lasting for 9 months, involving hands, wrists, elbows, shoulders and knees, which was unresponsive to non-steroidal anti-inflammatory drugs. Past medical history was remarkable for recurrent infections since early childhood, including recurrent bacterial RTIs, furunculosis, giardiasis and one episode of hypoxemic *Legionella pneumophila* pneumonia requiring invasive mechanical ventilation 3 years before admission. BCG vaccination was uneventful and there was no increased susceptibility to Herpesviruses, Human Papillomaviruses nor fungi. Family medical history was negative for recurrent infections, autoimmune nor lymphoproliferative disorders among first- and second-degree relatives (Fig. 1 A).

Clinical examination revealed splenomegaly (160 mm), present tonsils and no enlarged lymph nodes. The patient was afebrile and lung auscultation was normal. Blood cell counts were normal, and serum C-reactive protein level was moderately increased (24 mg/L). Blood cultures were sterile and whole-blood PCR screening was negative for HIV, HBV, HCV, parvovirus B19, SARS-CoV-2, EBV, CMV and enterovirus. Synovial fluid (SF) obtained from left knee arthrocentesis contained an inflammatory liquid with 9200 leukocytes/mm^3^ mostly composed of non-atypical lymphocytes. SF was sterile after a 10-day culture. No crystals were identified. X-rays of the involved joints showed no abnormality. Screening for auto-immune disorders including rheumatoid factor, antinuclear, anti-DNA and anti-cyclic citrullinated peptide antibodies was negative. Serum electrophoresis showed agammaglobulinemia confirmed by the absence of serum IgG, IgA and IgM. There was no detectable free light chain in the serum. Whole-body computed tomography revealed pan-sinusitis and bilateral bronchiectasis (Fig. 1B).

A primary immune deficiency was suspected. Peripheral blood lymphocyte phenotyping showed a normal number of T- and NK-cells but virtually no detectable B cells (0.2% CD19^+^). Percentage and number of naive and memory T cells were within normal range for age. Intracellular staining showed normal BTK expression in monocytes, ruling out XLA (Fig. 1 C, detailed in Supplementary Material). T-cell proliferation assays using cell tracer dilution were similar to control upon mitogen (Phytohemagglutinin (PHA), Pokeweed Mitogen (PWM) and anti-CD3/CD28) and candidin stimulations. Tuberculin stimulation was weakly positive, while tetanus toxoid stimulation yielded negative results, more than 20 years after last DTaP (Diphtheria-Tetanus-Pertussis) injection. Whole blood NGS encompassing 489 genes associated with monogenic IEI(including *BTK* and other autosomal-recessive agammaglobulinemia) was negative (detailed in Supplementary Material).

The IEI setting led to additional extensive microbiological work-up on SF: bacterial 16 S ribosomal RNA sequencing, molecular detection of *Mycobacterium tuberculosis complex* DNA and growth on Lowenstein media, fungi and parasite culture, molecular detection of *Tropheryma whipplei* DNA (in SF, blood and stools) and virus-specific real-time PCR including HSV-1/HSV-2, VZV, CMV, EBV, HHV-6, HHV-8, enterovirus and parvovirus B19 were all negative. In contrast, MNGS (described in [5]) performed on SF found 2.6 reads per million (RPM) matching human adenovirus (HAdV) sequences. No other infectious agent was detected. To further confirm and characterize HAdV infection, viral load and full genome were determined (detailed in Supplementary Material). Quantitative RT-PCR showed high levels of HAdV in SF (5.2 log_10_ copies/mL). Full genome sequencing was obtained with next generation sequencing enriched for HAdV sequences with a probe capture strategy (described in [6]) and identified HAdV C-1. A second knee arthrocentesis confirmed the detection of HAdV in SF (5.8 log_10_ copies/mL). HAdV replication was also present in plasma (2.9 log_10_ copies/mL) and stool (6.2 log_10_ copies/g) simultaneously. Nasopharyngeal sample was negative for HAdV and there was no respiratory involvement.

The patient received high-dose (2 g/kg every 3 weeks) human polyvalent intravenous immunoglobulins (IgG1 55-67%, IgG2 29-37%, IgG3 1-4%, IgG4 1-3%, IgA ≤22 µg/mL) with improvement of joint pain and swelling one week after treatment initiation. After the second infusion, the patient became totally asymptomatic. HAdV viral loads rapidly decreased in blood and stool samples and became undetectable 21 days after immunoglobulin initiation (Fig. 1D).

## Discussion

We here report a case of chronic HAdV-C1 polyarthritis diagnosed using MNGS in an immunocompromised patient.

We performed a review of the literature and identified two previous cases of HAdV-related inflammatory joint disease. The first patient (reported in 1974) was an otherwise healthy adult presenting with an acute systemic disorder including fever, rash, acute respiratory disease and bilateral synovitis, related to Adenovirus type 7 [7]. He recovered spontaneously. No screening for an immunodeficiency was performed. The second patient (reported in 1984) had a childhood history of recurrent RTIs before developing erosive polyarthritis at the age of 29 years [8]. Electron microscopy of a synovial specimen revealed intranuclear particles with adenoviral-like structures but full in-depth virologic characterization was not performed. Intra-articular crystals were also visualized. Interestingly, he was shown to have agammaglobulinemia with a normal B-cell count (CD19^+^=14% of total lymphocytes). No screening for a monogenic IEI was performed. He was lost to follow up and putative therapeutic options were not discussed. These findings expand the spectrum of viral susceptibility in patients with primary agammaglobulinemia.

HAdV-C1 causes benign respiratory, ocular, urinary tract or digestive infections in immunocompetent individuals, but may also cause severe and life-threatening infections in immunocompromised hosts [9]. Infections are usually disseminated with multiorgan involvement and high HAdV loads in stool (> 6 log_10_ copies/g) have been correlated to the risk of viremia and infection dissemination in allogeneic hematopoietic stem cell transplant recipients [10, 11]. The patient described herein had a chronic and long-lasting story of inflammatory joint disease with no other symptom, despite having high HAdV loads in the stool. Defenses against HAdV involve both T and B cell responses. B cells generate anti-HAdV antibodies with neutralizing capacities but do not protect from re-infection or re-activation. In contrast T cell immunity is critical to control HAdV replication and to prevent severe disease [3, 9]. The absence of overt T-cell defects may have played a role in the limited severity of HAdV-C1 infection in this patient, despite documented blood viremia. Further genetic investigations using whole-exome or whole-genome sequencing will be required to better characterize the underlying immune defect. This singular joint involvement somehow resembles the so-called “rheumatoid arthritis” lacking bone erosion and rheumatoid factor, that has been described in patients with primary agammaglobulinemia, and which improves after Ig replacement therapy [4, 12]. Most descriptions of this condition were published before year 2000, when in-depth viral studies were not routinely performed. This study suggests that adenoviral arthritis could be the cause of some inflammatory joint diseases encountered in patients with agammaglobulinemia, and that some other micro-organisms could be responsible for the remaining part of it. This also suggests that adenovirus-based live vaccines should be contraindicated in these patients. The present report also emphasizes the important role of MNGS, allowing the identification of novel infectious agents in patients with inborn or acquired immunodeficiencies. This could avoid potentially harmful immunosuppressive treatments and patients might instead benefit of high-dose Ig therapy.

## Electronic supplementary material

Below is the link to the electronic supplementary material.

Supplementary Material 1

## Data Availability

Detailed data and materials are available upon e-mail request to Dr. David Boutboul (david.boutboul@aphp.fr).
