# Supplementary material for: Human adenoviral (HAdV) chronic arthritis expands the infectious spectrum of primary agammaglobulinemia
Source: Virol J. 2022 Oct 31;19:172. doi: 10.1186/s12985-022-01905-z (PMC9623974; doi:10.1186/s12985-022-01905-z)
Supplement: Supplementary file 1 — Supplementary Material 1 [file 12985_2022_1905_MOESM1_ESM.docx]

**SUPPLEMENTARY MATERIAL**

*BTK intra-cellular staining using flow cytometry*

Immunophenotyping was performed on fresh-whole blood from the patient and a healthy control, with anti-CD14 Pacific Blue (BD Biosciences) and anti-CD19 FITC (Beckman Coulter) monoclonal antibodies, followed by intra-cellular staining using the PhosFlow Lyse/Fix Buffer, Phosflow PermWash Buffer I (BD Biosciences) according to manufacturer’s instructions, and the anti-BTK AlexaFluor 647 (clone 53/BTK) monoclonal antibody or corresponding mouse IgG2aK Alexa Fluor 647 isotype control. Acquisition was performed using a FACS Lyric cytometer (BD Biosciences), and data analyzed using FlowJo software (BD Biosciences).

*Whole-blood NGS of PIDs genes*

The exonic and flanking intronic regions (+/- 20bp) of 489 genes (listed below), implied in monogenic human inborn errors of immunity, were studied. Genomic DNA was extracted from a whole-blood EDTA sample. The captures by hybridization libraries (XT-HS2 method, Agilent technologies) were sequenced on an Illumina NextSeq500. After demultiplexing, sequences were aligned to the reference human genome hg18 (BWA). The mean depth of coverage was 844x, with 99.5% of sequenced regions >30x. Data were analyzed with the Genome Analysis Toolkit (GATK ; Haplotypecaller, Unifigenotyper, Samtools and Freebayes), following the Broad Institute best practices. The genetic variations were compared to gnomAD, HGMD and ClinVar genomic human databases, and the functional impact of the amino-acid substitutions was evaluated thanks to *in silico* prediction softwares Polyphen-2 Human Var, SIFT, Mutation Taster and CADD.

*List of sequenced genes*

The following genes were sequenced (genes associated with primary agammaglobulinemia are highlighted in yellow) :

| *ACD* |
| --- |
| *ACP5* |
| *ACTB* |
| *ACTN1* |
| *ADA* |
| *ADA2 (CECR1)* |
| *ADAM17* |
| *ADAR* |
| *AICDA* |
| *AIRE* |
| *AK2* |
| *ALPI* |
| *AP1S3* |
| *AP3B1* |
| *AP3D1* |
| *APOL1* |
| *ARHGEF1* |
| *ARPC1B* |
| *ATAD3A* |
| *ATG4A* |
| *ATM* |
| *ATP6AP1* |
| *B2M* |
| *BACH2* |
| *BCL10* |
| *BCL11B* |
| *BLM* |
| ***BLNK*** |
| *BPIFA1* |
| ***BTK*** |
| *C1QA* |
| *C1QB* |
| *C1QC* |
| *C1R* |
| *C1S* |
| *C2* |
| *C2orf69* |
| *C3* |
| *C5* |
| *C6* |
| *C7* |
| *C8A* |
| *C8B* |
| *C8G* |
| *C9* |
| *CACNA1F* |
| *CARD11* |
| *CARD14* |
| *CARD8* |
| *CARD9* |
| *CARMIL2 (RLTPR)* |
| *CASP1* |
| *CASP10* |
| *CASP8* |
| *CCBE1* |
| *CCDC28B* |
| *CCR2* |
| *CD19* |
| *CD247* |
| *CD27* |
| *CD28* |
| *CD3D* |
| *CD3E* |
| *CD3G* |
| *CD4* |
| *CD40* |
| *CD40LG* |
| *CD46* |
| *CD48* |
| *CD55* |
| *CD59* |
| *CD70* |
| ***CD79A*** |
| ***CD79B*** |
| *CD81* |
| *CD8A* |
| *CDC42* |
| *CDCA7* |
| *CDH17* |
| *CDH7* |
| *CEBPE* |
| *CFB* |
| *CFD* |
| *CFH* |
| *CFHR1* |
| *CFHR2* |
| *CFHR3* |
| *CFHR4* |
| *CFHR5* |
| *CFI* |
| *CFP* |
| *CHUK* |
| *CIB1* |
| *CIITA* |
| *CLPB* |
| *COPA* |
| *COPG1* |
| *CORO1A* |
| *CR2 (CD21)* |
| *CSF2RA* |
| *CSF2RB* |
| *CSF3R* |
| *CTC1* |
| *CTLA4* |
| *CTNNBL1* |
| *CTPS1* |
| *CTSC* |
| *CXCR2* |
| *CXCR4* |
| *CYBA* |
| *CYBB* |
| *CYBC1* |
| *DBF4* |
| *DBR1* |
| *DCLRE1B* |
| *DCLRE1C (ARTEMIS)* |
| *DDX58* |
| *DEF6* |
| *DGAT1* |
| *DIAPH1* |
| *DKC1* |
| *DNAJC21* |
| *DNASE1L3* |
| *DNASE2* |
| *DNMT3B* |
| *DOCK11* |
| *DOCK2* |
| *DOCK8* |
| *DSG1* |
| *EFL1* |
| *ELANE* |
| *ELF4* |
| *EOMES* |
| *EPG5* |
| *ERBIN (ERBB2IP)* |
| *ERCC6L2* |
| *EXTL3* |
| *FAAP24* |
| *FADD* |
| *FAS* |
| *FASLG* |
| *FAT4* |
| *FBLIM1* |
| *FCGR3A* |
| *FCHO1* |
| *FCN3* |
| *FERMT1* |
| *FERMT3* |
| *FLT3LG* |
| *FNIP1* |
| *FOXN1* |
| *FOXP3* |
| *FPR1* |
| *FUT2* |
| *G6PC3* |
| *G6PD* |
| *GAR1* |
| *GATA2* |
| *GFI1* |
| *GIMAP5* |
| *GIMAP6* |
| *GINS1* |
| *HAVCR2 (TIM3)* |
| *HAX1* |
| *HCK* |
| *HELLS* |
| *HMOX1* |
| *HYOU1* |
| *ICOS* |
| *ICOSLG* |
| *IFIH1* |
| *IFNAR1* |
| *IFNAR2* |
| *IFNG* |
| *IFNGR1* |
| *IFNGR2* |
| *IGHG1* |
| ***IGHM*** |
| *IGKC* |
| ***IGLL1*** |
| *IKBKB* |
| *IKBKG (NEMO)* |
| *IKZF1 (IKAROS)* |
| *IKZF2 (HELIOS)* |
| *IKZF3 (AIOLOS)* |
| *IL10* |
| *IL10RA* |
| *IL10RB* |
| *IL12B* |
| *IL12RB1* |
| *IL12RB2* |
| *IL13RA1* |
| *IL17A* |
| *IL17F* |
| *IL17RA* |
| *IL17RC* |
| *IL18BP* |
| *IL1RN (DIRA)* |
| *IL21* |
| *IL21R* |
| *IL23R* |
| *IL2RA (CD25)* |
| *IL2RB (CD122)* |
| *IL2RG (GAMMAC)* |
| *IL36RN* |
| *IL37* |
| *IL6R* |
| *IL6ST* |
| *IL7* |
| *IL7R* |
| *INO80* |
| *IRAK1* |
| *IRAK4* |
| *IRF1* |
| *IRF2BP2* |
| *IRF3* |
| *IRF4* |
| *IRF7* |
| *IRF8* |
| *IRF9* |
| *ISG15* |
| *ITCH* |
| *ITGAX* |
| *ITGB2* |
| *ITK* |
| *ITPKB* |
| *ITPR3* |
| *JAGN1* |
| *JAK1* |
| *JAK2* |
| *JAK3* |
| *KCNA5 (HCK1)* |
| *KDM6A* |
| *KMT2A* |
| *KMT2D* |
| *KPNA2* |
| *KRAS* |
| *LACC1* |
| *LAMTOR2* |
| *LAT* |
| *LCK* |
| *LCP2 (SLP76)* |
| *LIG1* |
| *LIG4* |
| *LIPA* |
| *LPIN2* |
| *LRBA* |
| *LSM11* |
| *LY9* |
| *LYN* |
| *LYST* |
| *MAGT1* |
| *MALT1* |
| *MAN2B2* |
| *MAP1LC3B2* |
| *MAP3K14 (NIK)* |
| *MAPK8* |
| *MASP2* |
| *MCM10* |
| *MCM4* |
| *MCTS1* |
| *MDFIC* |
| *MEFV* |
| *MOGS* |
| *MPO* |
| *MR1* |
| *MRE11* |
| *MRTFA (MKL1)* |
| *MS4A1 (CD20)* |
| *MSH6* |
| *MSN* |
| *MTHFD1* |
| *MVK* |
| *MYD88* |
| *MYSM1* |
| *NBN (NBS1)* |
| *NCF1* |
| *NCF2* |
| *NCF4* |
| *NCKAP1L* |
| *NCSTN* |
| *NFAT5* |
| *NFE2L2* |
| *NFKB1* |
| *NFKB2* |
| *NFKBIA* |
| *NFKBID* |
| *NHEJ1 (CERNUNNOS)* |
| *NHP2* |
| *NLRC4* |
| *NLRP1* |
| *NLRP12* |
| *NLRP3* |
| *NLRP6* |
| *NOD2* |
| *NOP10* |
| *NOS2* |
| *NRAS* |
| *NSMCE3* |
| *OAS1* |
| *ORAI1* |
| *OTULIN* |
| *PARN* |
| *PAX1* |
| *PAX5* |
| *PCNA* |
| *PDCD1* |
| *PEPD* |
| *PGM3* |
| *PI4KA* |
| *PIK3CA* |
| *PIK3CD* |
| *PIK3CG* |
| ***PIK3R1*** |
| *PLCG2* |
| *PMS2* |
| *PNP* |
| *POLA1* |
| *POLD1* |
| *POLD2* |
| *POLE* |
| *POLE2* |
| *POLR3A* |
| *POLR3C* |
| *POLR3F* |
| *POMP* |
| *POU2AF1 (BOB1)* |
| *PRF1* |
| *PRKCD* |
| *PRKDC* |
| *PSEN1* |
| *PSENEN* |
| *PSMA3* |
| *PSMB10* |
| *PSMB2* |
| *PSMB4* |
| *PSMB8* |
| *PSMB9* |
| *PSMG2* |
| *PSTPIP1* |
| *PTCRA* |
| *PTEN* |
| *PTPN2* |
| *PTPRC (CD45)* |
| *PYCARD* |
| *RAB27A (GS2)* |
| *RAC2* |
| *RAG1* |
| *RAG2* |
| *RAP1B* |
| *RASGRP1* |
| *RBCK1 (HOIL1)* |
| *RC3H1* |
| *REL* |
| *RELA* |
| *RELB* |
| *RFX5* |
| *RFXANK* |
| *RFXAP* |
| *RHOG* |
| *RHOH* |
| *RIPK1* |
| *RMRP* |
| *RNASEH2A* |
| *RNASEH2B* |
| *RNASEH2C* |
| *RNF168* |
| *RNF31 (HOIP)* |
| *RNU4ATAC* |
| *RNU7-1* |
| *RORC* |
| *RPA1* |
| *RPSA* |
| *RTEL1* |
| *SAMD9 (MIRAGE)* |
| *SAMHD1* |
| *SASH3* |
| *SBDS* |
| *SBNO2* |
| *SDHA* |
| *SEC61A1* |
| *SEMA3E* |
| *SEMA4D (CD100)* |
| *SERPING1* |
| *SH2D1A (SAP)* |
| *SH3BP2* |
| *SH3KBP1 (CIN85)* |
| *SHARPIN* |
| *SKIV2L* |
| *SLC11A1* |
| *SLC29A3* |
| *SLC35C1* |
| *SLC37A4* |
| ***SLC39A7*** |
| *SLC46A1* |
| *SLC7A7* |
| *SMARCAL1* |
| *SMARCD2* |
| *SNORA31* |
| *SOCS1* |
| *SP110* |
| ***SPI1 (PU.1)*** |
| *SPINK5* |
| *SPPL2A* |
| *SRP54* |
| *SRP72* |
| *STAT1* |
| *STAT2* |
| *STAT3* |
| *STAT5B* |
| *STIM1* |
| *STK4* |
| *STN1* |
| *STX11* |
| *STXBP2* |
| *STXBP3* |
| *SYK* |
| *TALDO1* |
| *TAP1* |
| *TAP2* |
| *TAPBP* |
| *TAZ* |
| *TBK1* |
| *TBX1* |
| *TBX21* |
| ***TCF3*** |
| *TCN2* |
| *TERC* |
| *TERT* |
| *TET2* |
| *TFRC* |
| *TGFB1* |
| *TGFBR1* |
| *TGFBR2* |
| *THBD* |
| *TICAM1* |
| *TIFA* |
| *TINF2* |
| *TIRAP* |
| *TLR3* |
| *TLR7* |
| *TLR8* |
| *TMC6* |
| *TMC8* |
| *TMEM173 (STING1)* |
| *TNF* |
| *TNFAIP3 (A20)* |
| *TNFRSF11A* |
| *TNFRSF13B (TACI)* |
| *TNFRSF13C (BAFFR)* |
| *TNFRSF1A (TNFAR)* |
| *TNFRSF4* |
| *TNFRSF9 (CD137, 4.1BB)* |
| *TNFSF12 (TWEAK)* |
| *TNFSF13 (APRIL)* |
| *TNFSF9* |
| *TOM1* |
| *TOP2B* |
| *TP53* |
| *TPP2* |
| *TRAC* |
| *TRAF3* |
| *TRAF3IP2* |
| *TREX1* |
| *TRIM22* |
| *TRNT1* |
| *TTC37* |
| *TTC7A* |
| *TYK2* |
| *UBA1* |
| *UNC13D* |
| *UNC93B1* |
| *UNG* |
| *USB1* |
| *USP18* |
| *USP43* |
| *VPS13B* |
| *VPS45* |
| *WAS* |
| *WASF2* |
| *WDR1* |
| *WIPF1* |
| *WRAP53* |
| *XIAP* |
| *XRCC4* |
| *ZAP70* |
| *ZBTB24* |
| *ZNF341* |
| *ZNFX1* |

*Adenoviral real-time PCR*

Adenoviral RT-PCR was performed using ADENOVIRUS R-GENE® (bioMerieux, Craponne, France)

*Metagenomic Next Generation Sequencing*

All processed samples were pretreated with bead beating. Total nucleic acids were extracted using Nuclisens EAsymag (Biomerieux) according to manufacturer’s protocol. We prepared DNA libraries with Nextera XT DNA, and RNA libraries with TrioRNAseq (Nugen) according to manufacturer instructions. Libraries were multiplexed with negative control (NTC) (water) and sequenced on an Illumina NextSeq using 150/150-bp paired-end sequencing.

A total of 28 million reads was generated and analyzed with our diagnostic pipeline (github). Briefly, after cleaning and deduplicating using TRIMMOMATIC and Dedupe, taxonomic assignment was carried out using Kraken2 with Viral, Bacterial and Human Refseq databases. Bacterial and viral assigned reads were verified using Blastn on reduced database. Coverage, Number of reads aligned and comparison with NTC were used for pathogen presence assessment.
